# Transmission of Stress-Induced Learning Impairment and Associated Brain Gene Expression from Parents to Offspring in Chickens

**DOI:** 10.1371/journal.pone.0000364

**Published:** 2007-04-11

**Authors:** Christina Lindqvist, Andrew M. Janczak, Daniel Nätt, Izabella Baranowska, Niclas Lindqvist, Anette Wichman, Joakim Lundeberg, Johan Lindberg, Peter A. Torjesen, Per Jensen

**Affiliations:** 1 IFM Biology, Linköping University, Linköping, Sweden; 2 Department of Animal and Aquacultural Sciences, Norwegian University of Life Sciences, Ås, Norway; 3 Department of Animal Environment and Health, Swedish University of Agricultural Sciences, Skara, Sweden; 4 School of Biotechnology, Department of Gene Technology, Royal Institute of Technology, Stockholm, Sweden; 5 Hormone Laboratory, Aker University Hospital HF, Oslo, Norway; Indiana University, United States of America

## Abstract

**Background:**

Stress influences many aspects of animal behaviour and is a major factor driving populations to adapt to changing living conditions, such as during domestication. Stress can affect offspring through non-genetic mechanisms, but recent research indicates that inherited epigenetic modifications of the genome could possibly also be involved.

**Methodology/Principal Findings:**

Red junglefowl (RJF, ancestors of modern chickens) and domesticated White Leghorn (WL) chickens were raised in a stressful environment (unpredictable light-dark rhythm) and control animals in similar pens, but on a 12/12 h light-dark rhythm. WL in both treatments had poorer spatial learning ability than RJF, and in both populations, stress caused a reduced ability to solve a spatial learning task. Offspring of stressed WL, but not RJF, raised without parental contact, had a reduced spatial learning ability compared to offspring of non-stressed animals in a similar test as that used for their parents. Offspring of stressed WL were also more competitive and grew faster than offspring of non-stressed parents. Using a whole-genome cDNA microarray, we found that in WL, the same changes in hypothalamic gene expression profile caused by stress in the parents were also found in the offspring. In offspring of stressed WL, at least 31 genes were up- or down-regulated in the hypothalamus and pituitary compared to offspring of non-stressed parents.

**Conclusions/Significance:**

Our results suggest that, in WL the gene expression response to stress, as well as some behavioural stress responses, were transmitted across generations. The ability to transmit epigenetic information and behaviour modifications between generations may therefore have been favoured by domestication. The mechanisms involved remain to be investigated; epigenetic modifications could either have been inherited or acquired *de novo* in the specific egg environment. In both cases, this would offer a novel explanation to rapid evolutionary adaptation of a population.

## Introduction

Stress will affect any species brought into captivity, because of human handling and restriction of behavioural possibilities [1]. It has probably been an important factor driving adaptation during domestication, a process which has produced an enormous phenotypic intraspecific variation in an evolutionary short time [2]. Selection experiments have shown that adaptation to stressful captivity conditions, such as reduced fearfulness, can occur in few generations, causing a rapid evolutionary process which simultaneously affects a wide range of animal behaviour and physiology [3], [4].

The mechanisms causing such rapid changes in the biology of a population remain elusive. However, recent research in various species suggests that stress in one generation may lead to long-term effects on the offspring. In plants, for example, exposure to stressful UV-light may cause an epigenetic trace which is maintained in many generations of offspring growing under non-stressful conditions [5]. In mammals and birds, stress experienced during pregnancy or egg formation may have wide-ranging effects on offspring phenotype [6], [7].

Behavioural changes occurring as a response to environmental challenges such as stress are normally considered not to be transmissible to offspring. As already mentioned, offspring phenotypes, including behaviour, are affected by the hormonal environment in eggs or in the uterus [8], [9], but the phenotypic plasticity emerging from such a system is thought to act through non-genetic mechanisms, and is therefore usually not considered to be inherited in the true sense of the word. For example, hormones could alter functions in the nervous system during embryogenesis, thereby influencing the behaviour of the offspring without evoking any detectable and lasting genetic effects [6].

On the other hand, epigenetic mechanisms have been shown to be involved in phenotypic plasticity within a certain generation. For example, the type of maternal behaviour shown by rat mothers changes epigenetic marking of important genes in the young, which in turn is associated with long lasting behaviour effects [10]. Furthermore, there is emerging evidence that epigenetic modifications acquired in one generation may sometimes be inherited by the offspring [5], [11], [12]. Although there is so far no evidence that such inherited epigenetic modifications of gene expression can be linked to specific behaviour, it is an emerging possibility which deserves to be investigated; if this could occur, it would provide a possible mechanism for rapid modification of behaviour in a population of animals facing environmental challenges, such as during domestication.

In the present work, we use ancestral red junglefowl (RJF) and domesticated White Leghorn (WL) chickens, selected for growth and egg production, to study whether stress-induced behaviour modifications in the ability to solve a spatial learning task was transferred to the offspring. Furthermore, we use a cDNA microarray to investigate whether the stress-induced learning impairment was associated with a modification in gene expression profiles of the hypothalamus and pituitary (brain regions central to the stress response), and whether a similar difference was seen in gene expression of offspring from stressed parents. If both these effects would occur simultaneously – both behaviour and gene expression differences in parents and offspring – this would indicate that stress responses acquired in one generation could affect the offspring by means of genomic effects, for example through inter-generational transfer of epigenetic modifications.

## Materials and Methods

### Animals, environments and treatment

We studied domesticated White Leghorn layers (WL) and red junglefowl (RJF), the ancestor of all domestic chickens [13]. The complete background of the animal lines has been described by Schütz et al [14]. The RJF used in the experiment were derived from a zoo population and had been kept in the research facility for four generations. The WL were from a selection line, SLU13, bred at the Swedish University of Agricultural Sciences. This line is specifically selected for egg mass.

Fifteen males and 15 females of each population were subjected to a chronic mild stressful treatment, which consisted of an unpredictable light-dark rhythm: controlling for total number of light hours per week, light and dark periods of 3, 6, 9, 12, 18 and 24 hours were randomly applied, whereas the same number of control birds always had a 12:12 h light:dark cycle. The total number of light hours per week were identical for the treatments. Treatment and control pens were situated in the same room, next to each other. The stress treatment, was commenced when the parents were five weeks old, and continued until the birds were 260 days old.

Birds in the unpredictable light regime were thus unable to predict when and for how long food and water would be available, since they usually never feed in darkness, and to predict when to settle on perches, where they normally spend the dark periods. In all other respects, the pens were identical for the treatments. The stress treatment did not significantly affect either growth rate or adult weight in any of the breeds, indicating that the birds were able to cope with the environment without adverse physiological consequences.

In the parental generation, where the stress treatment was applied, the newly hatched chicks of both populations were kept for three weeks in pens containing wood-shavings, heaters, feeders and water, and were fed commercial chicken feed *ad lib*. From the start of experimental treatment, the birds were kept in pens measuring 3×3 m, containing perches, wood shavings, feed and water. When the birds started to lay eggs, the pens were equipped with group nests on the floor. Room temperature was kept at about 22°C, and light levels at about 30 Lux. To balance for any pen effects, the groups were moved between pens every third week, without changing the light treatment for the group.

For the offspring studies, we collected in total 267 eggs from the four groups, equally distributed between the treatments and populations, and incubated these in the same Grumbach incubator with automatic control of temperature, humidity, and egg turning. There were no significant differences between breeds or treatment in hatchability of the eggs. Out of the hatched chickens, we randomly selected 34 chicks from each treatment and population for further experiments. At hatching, the chicks were weighed and marked with wing-tags, and vaccinated intra-muscularly with 0.2 ml Marexine vet® (against Marek's disease). The newly hatched chicks of each population were kept in one group with a 12/12 h light-dark rhythm regardless of parents, under the same pen conditions as described above for the parents.

### Learning test

We randomly selected 18 parental animals from each group (one group consisting of animals of the same breed and receiving the same treatment), and all offspring (17–19 per group; one group consisting of the same breed and whose parents had received the same treatment), for test of spatial learning ability. Parents were tested starting when they were 133 days of age, and offspring when they were 33 days old. Some red junglefowl were unable to locate the food at all in the first five tests, apparently due to being to stressed by the test situation, and were excluded from the analysis (Parents: control 4, stress 5; Offspring: stress 1). The learning test was conducted in a T-maze, following 15 h of feed deprivation (of which 12 h were in darkness). Each arm connected to the start box was 3.25 m long (1.6 m for the offspring), measured from the opening of the start box, and 0.5 m wide, and at the end of each arm was a 90° turn left or right, followed by another 1.0 m (0.8 m for the offspring) long arm. During the test period, which lasted for a maximum of two weeks for any individual, all birds were kept in pens with 12/12 h light-dark cycles, so the tests could be carried out at the same time of the day for birds from both treatments. The birds were habituated to the arena during 40 min in groups of 6 birds, and during this habituation time there was food available in both ends of the maze. When tested, a single bird was placed in the start-box when the room was dark, and at the start of each test, the light was turned on and a guillotine door opened to allow the bird access to the arena. Food was situated at the far end of one of the arms, out of the bird's sight. For a particular bird, the food was always situated in the same end of the maze, and the direction was balanced between breeds and treatments. Each test session lasted for a maximum of 10 min, or until the bird found the food and had fed for one minute. The animals were tested twice per day with an interval of one hour between test sessions, and for each individual, the feed was always situated in the same arm.

The behaviour was recorded from a distance with the help of video cameras. The first choice of arm was recorded on each test session, and the bird was considered to have made a choice when it entered the end alley of any of the arms. When a bird chose the correct arm first on five out of six consecutive test sessions, it was considered to have solved the task, and was returned to its home pen. We counted the choices starting from the test session where the bird first located the food, so if a bird initially did not find any food at all, these test sessions were disregarded in the analysis.

### Competition test

At three weeks of age, the capacity of the offspring to compete for food was determined by entering them two at a time (same population, but parents had different treatments) into a pen containing a single feed container. The feeder had a narrow opening, through which only one chick could feed at a time. During five minutes from the time when the chicks started feeding, the duration of time in which each individual occupied the feeder was recorded.

### Corticosterone analysis

Approximately 10 ml of yolk and albumen was collected from each of five eggs from each of the treatments, and frozen immediately after sampling. The levels of corticosterone in the samples were analysed using the radioimmunoassay (RIA) described by Lofthus et al.[15]. Prior to assay, 0.5 g of the thawed chicken egg yolk and albumen were extracted with diethylether. A specific corticosterone antiserum was used in the RIA (Cat. No. 07-120016, ICN, Irvine, CA, USA). The intra-assay coefficient of variation was 8.0% and the inter-assay coefficient of variation was 10.0%. The lower limit of detection for the analysis used in the present study was 0.2 ng/ml albumen or yolk.

### Tissue collection, RNA isolation and cDNA synthesis

In total 32 birds, 16 from each generation (two males and two females from each group of animals), were killed and their brains immediately removed. Hypothalamus and pituitary were collected from each brain and snap-frozen in liquid nitrogen and then stored at −80°C until RNA isolation. The frozen hypothalamus and pituitary were merged and homogenized in TRIzol®. Total RNA was isolated using the TRIzol® (Invitrogen) procedure with 1 ml TRIzol® to every 50 mg of tissue. The manufacturer's standard protocol was used, with the exception of adding 0.25 ml isopropanol and 0.25 ml 0.8 M disodium citrate solution in the RNA precipitation step. Ten microgram total chicken RNA was used for cDNA synthesis and then labeled with Cy3 or Cy5 mono-reactive dye (Amersham biosciences) and cDNA was synthesized and purified using a protocol developed at the Royal Institute of Technology (www.ktharray.se, protocol SOP 002).

### Microarray hybridization and analysis

To examine the gene expression profiles in the selected tissues, we used a cDNA microarray with 13907 cDNA clones. The microarrays were based on a testis and brain library from the same populations of RJF and WL as used in the present experiment [16]. For hybridization to the microarray, we used the protocol SOP 003, developed at the Royal Institute of Technology, and available at the same web-address as above. Details regarding this process have been described earlier by Fitzimmon [17]. Because of the low yield of total RNA the samples were amplified using the RiboAmp™ RNA Amplification Kit 0201 (Arcturus). The quantity and quality of the RNA was assessed using a NanoDrop® spectrophotometer (NanoDrop Technologies Inc) and the Agilent 2100 Bioanalyzer (Agilent Technologies).

The microarray experiment was set up as a reference design, using a RNA pool of all individuals in each generation as reference. Data analysis was performed in a R-software environment (freeware version 2.2.0) using the KTH package as described by Fitzimmons [17]. After filtering (using the strictest filter criteria), we had a total of 9033 spots with data from all samples.

The analysis compared the expression levels of every single spot on the microarray relative to a reference sample, and then compared the relative expression levels between stressed and control birds, or between birds whose parents had received stress or control treatment. Differential expression (DE) of a spot therefore indicates that the gene represented by the spot was up- or downregulated by the treatment, or – in the offspring – by the treatment of the parents.

We considered a gene to show differential expression (DE) if both the magnitude and the probability of the difference in expression level between stressed and control animals (or their offspring) were high. In previous studies using this microarray [17], the criteria has been that the M value>1 (the M-value is the log_2_ of the difference in expression level, so M = 1 means that there was a two-fold change in expression level), and B>0 (B = log odds ratio of expression; the B-value estimates the certainty of DE vs non-certainty of DE, and includes correction for false discovery rate).

We further calculated the correlation between DE of the genes in the parents and in the offspring. The majority of the microarray spots had an M-value of around 0, which, together with the fact that we compared large datasets with over 9000 measurements, might inflate the p-value of the correlation coefficient and distort a statistical comparison between the generations. Therefore, we used a randomization procedure to test the breed difference in the transgenerational transfer of DE. We generated 50 different datasets from each of the populations and treatments, each consisting of 500 randomly selected spots on the microarrays (approximately 5% of the total number of spots in each dataset). For each of the datasets, we calculated the correlation coefficient between the M-values of the included spots in the parents and the corresponding M-values in the offspring, and then estimated the mean correlation coefficient and its 95% confidence interval in this set of random samples. We could then use a standard t-test to compare the r-values between populations.

### Real-time RT-PCR verification

In order to verify the microarray results with a different method, we selected nine genes showing high M- and B-values. Non-amplified total RNA was used in Real-time RT-PCR using TaqMan® Reverse Transcription reagents and Power SYBR® Green Master Mix (Applied Biosystems). The procedure followed the manufacturer's recommendations except for an additional treatment with RNase free DNase (Novagene) described by Fitzimmons [17]. Primers for the nine differentially expressed genes on the microarrays were designed using Primer Express (Applied Biosystems) and Primer 3 [18] software. Real time PCR was performed on an Applied Biosystems 7500 fast Real-time PCR system. β-actin was chosen as an endogenous control after we discarded GAPDH due to low primer efficiency. Dissociation curves of all other reactions suggested that no primer dimers or secondary primer structures were present. Relative log_2_-difference in expression between stress and control treatments was calculated with the method developed by Pfaffl [19].

## Results and Discussion

**Figure 1:**
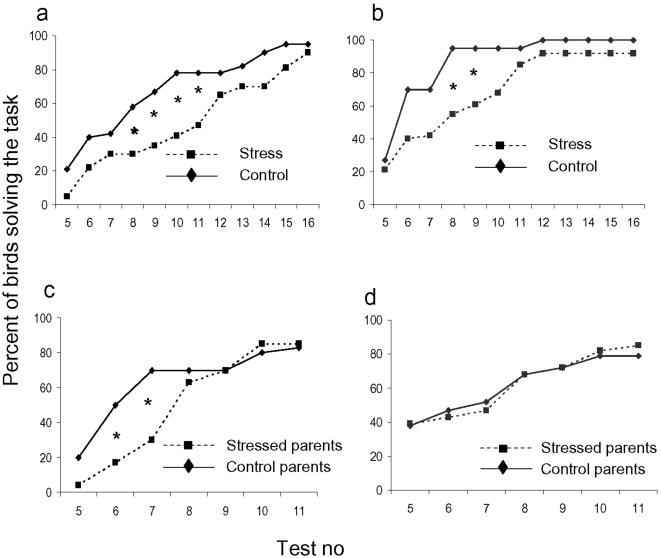
Spatial learning in White Leghorn parents and their non-stressed offspring. Each panel shows cumulative proportion of tested birds which had solved the spatial learning task at successive test instances; the criterion for solving the task was five correct choices out of six successive tests, so the smallest number of required tests was five. a, White Leghorn parents. b, Red junglefowl parents. c, White Leghorn offspring. d, Red junglefowl offspring. The differences in cumulative proportions of birds from different treatments solving the task were tested with χ^2^ –analysis after five test rounds and onwards, and significant differences are indicated (p<0.05).

In the spatial learning test, stressed parental birds of both populations took more tests to reach the solving criteria than controls (fig 1 a and b). There were significant effects of both breed (ANOVA, F_1,59_ = 7.4, p<0.01) and treatment (F_1,59_ = 6.5, p<0.05) on the average number of attempts needed to solve the task, where RJF and unstressed birds solved it faster. In addition, we tested the difference in the cumulative proportion of birds solving the test after each successive test round, and this analysis showed that the effect of treatment was mainly due to a slower acquisition in the stressed birds during the first part of the test series (fig 1 a and b). These results show that the stress treatment significantly affected the learning capacity of birds from both populations, and that RJF seemed to be overall better in the spatial learning task. This is not surprising, given the fact that WL have been selected for many generations in an environment with minimal stochasticity, whereas the natural environment of RJF probably has strongly favoured animals with a high capacity to locate and remember food sites.

When the offspring were tested in a similar, but smaller, T-maze as used for the parents, there were no significant effects of either breed or treatment (of the parents) on the average number of attempts needed to solve the task in an ANOVA-analysis. However, when again testing the difference in cumulative proportion of birds solving the test after each successive test round, chicks from stressed WL-parents were slower than offspring of control birds, whereas there was no difference in RJF (fig 1 c,d). Hence, although less pronounced than in the parents, offspring of stressed WL had a reduced ability to learn the spatial task compared to the offspring of non-stressed parents. This effect was not seen in RJF. The offspring were in general faster than the parents in learning the task. This could possibly be attributed to age differences in learning or to the fact that the arena was considerably smaller for the chickens.

RJF of stressed parents were significantly heavier than those of control parents at hatch, but WL offspring of stressed parents were significantly heavier than those of control parents at 8 days, and were also more competitive in a pairwise feed competition test at 27 days of age (Table 1). Similar to the results from the spatial test, there were no effects of parental treatment on competitive ability in RJF. It is likely that the higher weight gain in WL offspring of stressed parents was caused by their increased feed competition ability. In summary, the data suggest that the behaviour and weight of the WL offspring were modified as a result of the parents' experiences of a stressful environment.

**Table 1:** Weight and food competition capacity in offspring

	WL		RJF	
	Stressed parents	Control parents	Stressed parents	Control parents
Hatching weight (g)	44.4±0.5^a^	43.7±0.5 ^a^	26.4±0.5 ^b^	24.5±0.5 ^c^
8 days weight (g)	70.6±1.2 ^a^	66.0±1.2 ^b^	47.7±1.2 ^c^	44.8±1.2 ^c^
Percent time occupying feeder	58.2±5 ^a^	41.8±5 ^b^	51.0±5 ^c^	49.0±5 ^c^

Birds were weighed within an hour after hatching and at eight days of age. Food competition capacity was estimated as the percentage of time in which each individual occupied the feeder in a pair-wise competition test. The data were analysed with ANOVA, using breed and parental treatment as fixed independent variables. Data (LS Means±SEM) with different superscripts in the table differ significantly at p<0.05.

To examine the possibility that the behavioural effects were caused by stress related deposition of steroids in the eggs, we examined the levels of corticosterone in yolk and albumen of five eggs collected from different hens in each of the four groups. There was a significant effect of breed on albumen corticosterone levels (mean±SEM; RJF: 1.38±0.12 ng/ml, WL: 0.88±0.1 ng/ml; ANOVA, F_1,21_ = 38.7, p<0.001), but no effect of treatment, and no effects were observed of either treatment or breed on yolk levels. As the albumen levels reflect the blood levels during albumen formation in the uterus [20], i e, during a continuous period of about 10–12 h preceeding egg laying, the stress treatment as such did not seem to affect baseline adrenal corticosterone secretion. We are aware that other steroid hormones, not measured in this experiment, might have been affected by the treatment. The mentioned effects of treatment on hatching weight in RJF may indicate that the egg environments differed in some respect not measured here (Table 1).

**Figure 2:**
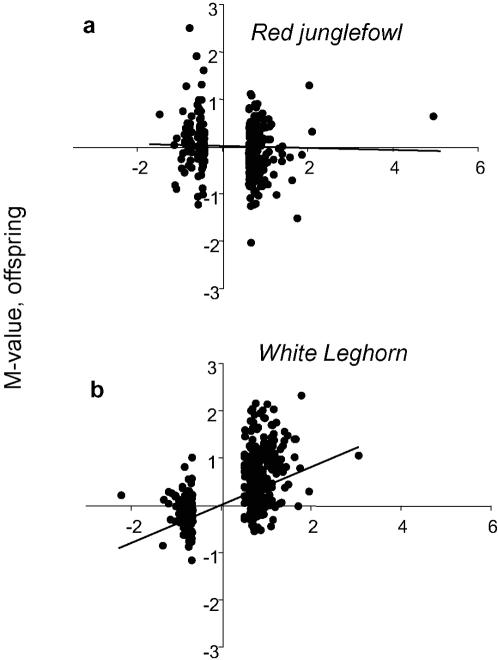
Correlations between magnitude of differential expression of genes between parents and offspring. Diagrams show M-values for the differential expression (comparing stressed vs control parents, and offspring of stressed vs offspring of control parents) of the 500 most differentially expressed genes (largest log_2_ difference caused by stress in parents) in (a) red junglefowl, and (b) White Leghorns. Each point represents one spot on the microarray. Positive M-values indicate upregulation and negative downregulation by stress (or by having stressed parents). The average correlation line is shown in both comparisons.

We hypothesized that the stress induced behaviour effects were associated with simultaneous changes in gene expression in hypothalamus or pituitary, which are brain regions central to the stress response [7]. If the differential expression induced by stress in the parents was transferred to the next generation, we expected a correlation between the DE of a gene in the parents and the DE of the same gene in the offspring; if a gene was upregulated by stress in the parents, the same gene should be upregulated in offspring of stressed chickens compared to offspring of non-stressed birds. A significant correlation between parent and offspring M-values was found in WL, but not in RJF, both when taking all the microarray spots into account and when only considering the 5% with the highest M-values (fig 2). For WL, the mean correlation coefficient (r) and its 95% confidence interval, r = 0.23±0.02, and for RJF r = 0.0006±0.1. The difference between these correlations was significant (p<0.001, t-test). This indicates that the regulatory change induced by stress was transferred to the offspring in WL, while there was no transfer between generations in RJF. Ten of the genes found in the top list (highest M-values) of both WL generations are listed in Table 2, along with their presently known annotations.

**Table 2:** Genes differentially expressed both in White Leghorn parents and offspring.

M-value		Description
Offspring	Parentals	
2.40	-1.18	RIKEN cDNA 4733401D09 (*Mus musculus*)
2.13	1.03	YFV MHC class I antigen (*Gallus gallus*)
2.01	1.17	Mitochondrial ribosomal protein L19 (*Gallus gallus*)
2.01	1.17	Not available
1.69	1.72	Not available
1.69	1.12	Not available
1.69	1.14	Matrix metallopeptidase 27 (*Gallus gallus*)
1.52	1.38	DNA for the terminal heterochromatic region (*Gallus gallus*)
1.50	1.17	Laminin alpha 3 subunit precursor (*Homo sapiens*)
1.47	1.48	Gamma-aminobutyric acid (GABA) receptor, rho 2 (*Gallus gallus*)

The table shows the 10 genes with highest differential expression in WL offspring, out of those which were among the 100 most differentially expressed genes in both WL parents and offspring. Negative M-values indicate that the expression level was higher in control than in stress birds.

According to M- and B-values, stress treatment of the parents was associated with significant expression differences in a number of genes in both generations, and there were more stress-induced DE genes in male parents than in females (Table 3). In the WL offspring of both sexes merged, the treatment of the parents affected the expression levels of several genes, whereas there were no such effects in the corresponding comparison of RJF offspring.

**Table 3:** Numbers of genes in parents and offspring brains showing differential expression caused by stress applied to parents.

	RJF			WL		
	Fathers	Mothers	Offspring	Fathers	Mothers	Offspring
M>1 and B>0	38	0	0	31	1	31
M>1 and B<0	209	66	154	360	182	155
M<1 and B>0	41	0	0	32	1	38

M-value is the log_2_ of the difference in expression level, and B-value is the log odds ratio of expression levels; the B-value estimates the certainty of DE vs non-certainty of DE. Common criteria for significant expression is that M>1 and B>0.

To verify the results from the microarray analysis, nine genes with high M- and B-values were selected for real-time RT-PCR analysis of the same tissues from the same individuals, and this analysis showed a corresponding DE of six of these based on their M-values (Table 4). This verification level is typically found for true expression differences using our cDNA microarray [16], supporting the interpretation that stress in parents caused a modification in the gene expression patterns in the hypothalamus and pituitary of both the parents and their offspring.

**Table 4:** Real-time RT-PCR data for nine genes selected for verification of microarray results

Gene name	M-value	Direction of regulation same as in microarray	Description
–	1.51	yes	Not availble
MMP271	0.00	–	Matrix Metalopeptidas (*Gallus gallus*)
NFKB	–0.48	no	Nuclear factor (*Gallus gallus* )
SOX18	–0.46	no	Transcription factor (*Gallus gallus*)
RLX1	1.98	yes	Putative 60S ribosomal protein (*Homo sapiens*)
FBXO32	2.96	yes	F-box only protein 32 (*Rattus norvegicus*)
–	2.06	yes	Hypothetical protein MGC13096 (*Homo sapiens*)
–	4.97	yes	Hypothetical protein XP_152521 (*Mus musculus*)
BDNF	1.63	yes	Brain-derived neurotrophic factor (*Gallus gallus*)

Because of the low number of biological replicates, no valid p-values could be estimated, so a gene with M>1 was considered to be differentially expressed in this analysis. All genes with M>1 showed the same direction of regulation (up or down-regulated by stress treatment) as on the microarray. The nine genes were selected based on their high M- and B-values in the microarray analysis. Relative expression levels (M-values) were estimated using β-actin as an internal control. Negative M-values indicate that the expression level was higher in control than in stress birds.

When considering the behavioural and the genetic data together, we conclude that both modified offspring behaviour and altered gene expression in offspring was seen in WL, but none of them in RJF. Although not conclusive from the present data, this may indicate that the alterations in gene expression could have been causally related to the alterations in behaviour. It also opens the possibility that domestication may have selected animals with an increased capacity to respond to environmental stress by affecting offspring phenotypes in captivity. Whereas the impairment in learning ability is difficult to explain from an adaptive perspective, the increased food competition ability of the offspring appears adaptive in an environment where food availability is unpredictable.

There are at least two possible mechanisms for the phenomenon suggested by our data. Firstly, altered epigenetic marking of specific genes resulting from chronic stress may have been transmitted directly to the offspring. This would require that the epigenetic markings were not erased at meiosis. As has been pointed out recently, there are a number of documented cases in vertebrates where such epigenetic marking (epialleles) is preserved across generations, leading to so-called “soft inheritance” of acquired traits [12]. Secondly, the epigenetic marking may have been acquired *de novo* in the egg, for example through the actions of stress-related steroid hormones deposited by the mother[6]. The mechanisms whereby this could create a correlation between the DE in the two generations is not known.

Apart from these options, the fact that the gene expression of male parents was more affected than that of females opens the possibility for paternal effects as well, the mechanisms of which remain unknown. Furthermore, since RJF had significantly higher albumen corticosterone levels, it is possible that this may actually have prevented the transfer of epigenetic marking by suppressing the up- or down-regulation of stress related genes in prehatching chickens; again the possible mechanism behind such an effect is not known.

Regardless of the mechanism for the transmission of genetic and phenotypic differences acquired in one generation, the evolutionary consequences of this process may be considerable.
